# The Resistance Force of the Anterior Cruciate Ligament during Pull Probing Is Related to the Mechanical Property

**DOI:** 10.3390/bioengineering9010004

**Published:** 2021-12-23

**Authors:** Takehito Hananouchi, Tomoyuki Suzuki, Erik W. Dorthe, Jiang Du, Darryl D. D’Lima

**Affiliations:** 1 Medical Engineering Laboratory, Department of Mechanical Engineering, Faculty of Engineering, Osaka Sangyo University, Daito 574-8530, Osaka, Japan; 2 Shiley Center for Orthopaedic Research and Education at Scripps Clinic, La Jolla, CA 92037, USA; dorthe@scripps.edu (E.W.D.); ddlima@scripps.edu (D.D.D.); 3 Department of Orthopaedic Surgery, Sapporo Maruyama Orthopedics Hospital, Sapporo 060-0007, Hokkaido, Japan; tomoyuki-s@r.sannet.ne.jp; 4 Department of Radiology, University of California San Diego, San Diego, CA 92093, USA; jiangdu@ucsd.edu

**Keywords:** probing device, mechanical property, anterior cruciate ligament

## Abstract

There are various methods for reconstructing the anterior cruciate ligament (ACL) from other muscles or tendons. Initial tension of the reconstructed ACL is one of the key elements affecting postoperative outcomes. However, tension cannot be measured after graft fixation. The only intraoperative assessment is pull probing, which is performed by pulling joint soft tissues with the arthroscopic probe and can be measured quantitatively. Therefore, its value might be used as an alternative value for the mechanical property of the ACL. Using a probing device one author developed to measure the resistance force of soft tissues quantitatively while probing, we measured the resistance force of dissected ACLs and used tensile testing to investigate the correlation between the resistance force and the mechanical property of the ligaments. According to the results, when a certain amount of tension (strain; 16.6%) was applied, its mechanical properties were moderately correlated (*r* = 0.56 [*p* = 0.045]) with the probing force. Therefore, the tension of the reconstructed ACL after fixation under real ACL reconstruction surgery can be derived from the value of the probing device.

## 1. Introduction

Current practice in the management of an anterior cruciate ligament (ACL) tear is to perform its reconstruction using a portion of other muscles or tendons [1,2,3,4,5]. Although there are various methods of ACL reconstruction, current research aims to develop a method of recreating or functionally substituting the original ACL [1,2,3]. An obstacle is that the mechanical properties of a reconstructed ACL are unclear. One approach for determining these mechanical properties is tensing the reconstructed ACL to confirm whether the reconstructed ligament works properly; however, there is no consensus on how much initial tension as a dynamic stabilizer of the knee joint must be applied. As discussed in [6,7,8], its evaluation is imprecise. The current technique for evaluating initial tension is to pull the reconstructed ligament with an arthroscopic probe [9], in a movement called “probing,” which is then assessed based on the surgeon’s intrinsic sensory feedback, or perception, of the amount of tension [10].

Most of the mechanical properties of ACLs are obtained through traditional material tests, such as tensile testing using fresh cadaveric tissues [11]. However, there have been no reports investigating the relationship between the mechanical property and tension assessment by probing. The probing device developed by one of the authors is capable of quantitatively evaluating the resistance force of joint soft tissues while probing [12], and properties of the hip labrum have been quantified using the probing device [10,13]. Therefore, using this device in ACL reconstruction surgery can clarify mechanical information regarding the ACL, such as its strength and/or stability, while probing. If we could determine the relationship between the parameters obtained using this device and during tensile testing, it might enhance the precision of probing evaluations.

The purpose of this study was to investigate whether there was a relationship between the mechanical properties of the ACL as calculated by the conventional tensile test and the resistance force of the ligament as measured by the novel probing device. For this purpose, we performed tensile tests to investigate the mechanical property of dissected ACLs. Immediately after those tests, pull probing of the ACLs was performed using the novel probing device.

## 2. Materials and Methods

ACL specimens were obtained from thirteen fresh-frozen knee cadaveric joints from eleven male and two female donors (mean: 50.9 years old; range: 25–96 years old at the time of death). Frozen knee joints were provided by a nonprofit whole-body donation company (United Tissue Network, Phoenix, AZ, USA). The mean length of the specimens was 25.1 mm (standard deviation [SD] 2.9 mm; range 20.5–29.6 mm). The following preparation of the specimens was performed.

The anterior–posterior plane of each ACL specimen was identified by two authors (TH and TS) to unify the direction when pull probing. After this step, the initial mechanical properties of each specimen were measured using a compression tester (Softgram, Shinko Denshi Co., Ltd., Tokyo, Japan) [14,15] to avoid collecting excessively degenerated specimens.

When the tip of the sensor was pressed against an object, the 3 mm-diameter indenter in the middle indented the ligaments at a depth of 0.5 mm, which enabled the built-in tuning fork pressure sensor to measure the softness of the ligaments. The tuning fork pressure sensor used the natural frequency of the ligaments to detect changes in the frequency due to the load and convert it into weight. Finally, it was possible to display Young’s modulus (Pa) based on Hertz’s elastic contact theory [16]. After the initial mechanical property of the ligaments was measured, the thickness of each ligament was measured using a caliper. Then, the width of each ligament was trimmed to 7 mm to correspond to the width of the adjusted tensile tester grippers.

**Figure 1:**
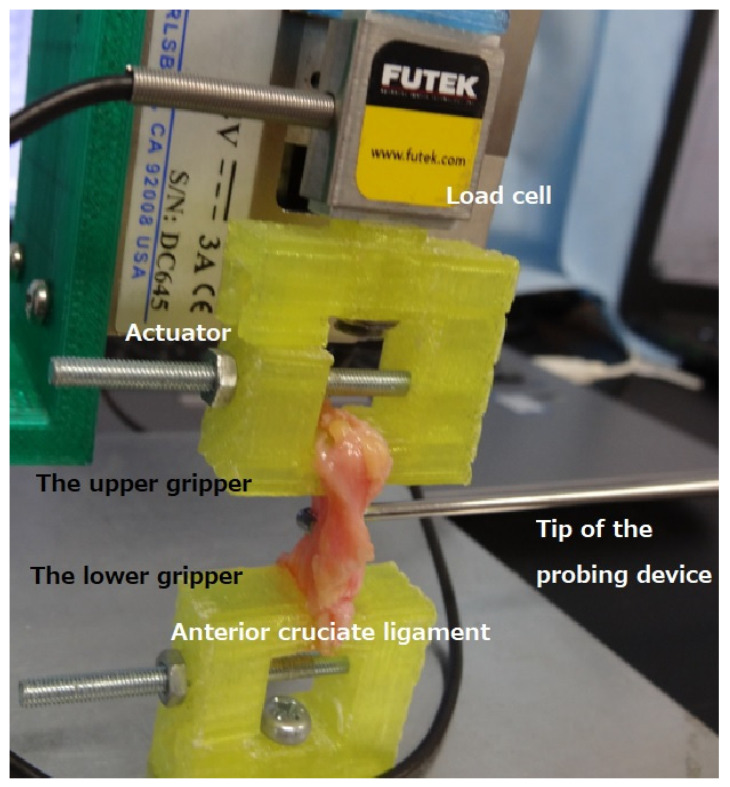
The tensile tester used in this study was an in-house-developed benchtop uniaxial loading device. Immediately after tension, pull probing horizontally to the anterior cruciate ligament specimen was performed using the probing device.

The tensile mechanical test was performed using an in-house-developed benchtop uniaxial loading device (Figure 1). The actuator and load cell were the same as those of the compression device used in previous studies [12,17]. A rack and pinion stage (TAR-38403L-M6, Sigma Koki Co., Ltd., Tokyo, Japan) connected the actuator and the base, which enabled adjustment of the position of the actuator in a vertical direction. ACL specimens were mounted on the device’s two grippers. ACL specimens were mounted on the device’s two grippers. The upper gripper was fixed to the actuator, whereas the lower gripper was fixed to the base of the device. The original distance between the two grippers was set to 15 mm. The software developed by one of the authors was adapted from LabVIEW software (version 17 of 2, National Instruments, Austin, TX, USA), and enabled the position of the actuator to be controlled in increments of 1 μm. Preloading was set to straighten the specimens (steps of 0.005 N were applied for 2 s periods up to the time the sample was held at 0.02 N). The speed of the actuator for this tensile test was performed at 4 mm/min rate. The maximum force was measured at three progressive displacements (1000, 2000, and 2500 μm). The stiffness and Young’s modulus in each ligament were calculated from the maximum force. With the grippers held at displacement, the resistance force of the ACL under the novel probing device simulated the typical probing approach under arthroscopy, i.e., the probe was hooked at the posterior plane of each ligament, and then the ligament was pulled out anteriorly by the probe.

The probing device consists of a probe component with a half-length size (200 mm) of a normal arthroscopic probe as well as a grip component in which the strain gauge sensor is embedded to measure the resultant force of three axes at the tip of the probe [12]. The detailed specifications were described in a previous paper [12]. Using the sliding aspect with a strain gauge sensor in the probing device, each ACL specimen was pulled vertically to its long axis by one author (TH) (Figure 1). The pulling distance was set to 3 mm in this study, following the method used in previous studies [10,12]. Dedicated software visualized and recorded the three forces in separate graphs in x, y, and z directions (x was the transverse direction; y was the vertical direction (direction of the hook); z was the probe axis). The software controlling the tensile tester recorded the increased axial force during pull probing. Each measurement by the probing device was taken three times. The averaged value of the three times was adopted.

### Statistical Analysis

Using the data obtained in this study, we examined each phase (the tensile distance was 1000 [1st], 2000 [2nd], and 2500 μm [3rd]) to determine whether there was a correlation between the stiffness and Young’s modulus in the tensile test, and the maximum force measured by the probing device during pull probing. The forces measured by the probing force were analyzed in the z direction, the resultant z and y directions, and the resultant three directions (x, y, and z). The effect size of r was determined as follows: 0.10 = small effect, 0.30 = medium effect, and 0.50 = large effect [18].

## 3. Results

The initial mechanical property of the compressive stiffness of the dissected ACL was the mean 7.5 kPa (SD, 5.2 kPa; range, 3.0–12.9 kPa) on the anterior plane and mean 6.1 kPa (SD, 2.2 kPa; range, 3.5–8.2 kPa) on the posterior plane. The anterior value tended to be greater than the posterior value. No specimen contained an excessively degenerative portion. Measurements obtained with the tensile tester are presented in Table 1.

Correlations between the stiffness and the probing force at each phase in the z direction, the resultant z and y directions, and the resultant three directions are shown in Table 2. According to the results, correlations between the stiffness and the probing force were significant only at the third phase, and the values were large. Correlations between the stiffness and the probing force at the third phase in the z direction, the resultant z and y directions, and the resultant three directions were 0.54 (*p* = 0.11), 0.53 (*p* = 0.049), and 0.56 (*p* = 0.045), respectively. The standard deviation of the three times at each time was, on average, 0.05 N at the first phase, 0.08 N at the second phase, and 0.09 N at the third phase.

The amount of increased force by the load cell of the tensile tester during pull probing was 0.48 N (SD; 0.12 N) in the first phase, 0.54 N (SD; 0.16 N) in the second phase, and 0.54 N (SD; 0.14 N) in the third phase.

## 4. Discussion

The aim of this study was to clarify whether the mechanical properties of the ligament after fixation are a contributing factor for evaluating the surgical outcome of ACL reconstruction. In this context, we investigated whether the resistance force of the dissected ACL during pull probing was related to its mechanical properties.

The results indicated that when there was weak tension during probing, there was no correlation between the resistance force and the probing force; however, when a certain amount of tension (strain 16.6%) was applied, its mechanical properties correlated with the probing force. Although there have been reports of probing after ACL fixation to check the stability and/or the condition [9,19], there were no detailed explanations of how much to pull and no reports clarifying the relationship with mechanical properties.

There have been several reports on the use of ACL tensile testers to investigate mechanical properties [11,20,21]. In general, it is known that the stress–strain curve of the ACL is nonlinear, with a loose, almost straight line up to a certain percentage of strain, and a tighter curve thereafter [11]. In one report, the boundary was in between 5 and 10%, which was similar to the present results [11]. The area up to this boundary is called the toe region [11], wherein the tissue is partially retentive while under tension. Since the amount of increased force by the load cell of the tensile tester at both the second and third phases was almost the same, we assume that the end of the toe region area is located around the second phase. The third phase crossed the toe region, which indicated that the mechanical properties and force were significantly correlated.

The clinical relevance of the findings of the current study connects the three following concepts. First, as mentioned in the background, various ACL reconstructions have been performed so far [1,2,3,4,5,6,7,8], and some reports have attempted to measure the tension of reconstructed ACL to find intraoperative measurements that lead to favorable postoperative outcomes [6,8]. Regardless of the method used, it is possible to measure the tension just before fixation, but not possible to determine the amount of tension after fixation. One of our authors indicated that, in terms of biomechanics, a triple-bundle method is better than using other approaches [6], one reason being that the laxity match pre-tension (LMP), which is graft tension to acquire joint stability of the intact knee, was less than that of other approaches [6,22]. In other words, if the tension is too strong, high tension can apply high pressures to the joint surface, which results in damage to the cartilage. Since the mean LMP in the triple-bundle reconstructed knees was 1.7 N for the anteromedial–medial bundle, 1.7 N for the anteromedial–lateral bundle, and 3.4 N for the posterolateral bundle, [6], we confirmed the tension of the reconstructed ACL under real ACL reconstruction surgery using the probing device, because the tension of the reconstructed ACL was stronger than the values of the probing force at the third phase. In order to investigate how much the tension of the reconstructed ACL is necessary, the measurement of the tension of original ACL should be performed under clinical knee arthroscopy cases, in which ACL is not damaged (e.g., meniscectomy) as one of the further studies.

Second, in total knee arthroplasty (TKA), the superiority between the posterior cruciate-retaining and the posterior cruciate-substituting designs is controversial [23,24]; the force value exerted by the probing device might distinguish the two TKA methods. In addition, an approach to preserving both cruciate ligaments was achieved in TKA, yet the clinical results were unfavorable [25,26]. However, intraoperative evaluation of both ligaments by the probing device might make it easier to assess the method used for TKAs.

Third, when considering a new regenerative medicine that substitutes original tissues, such as with artificial tendons or a biomaterial [27,28], a clinical trial is mandatory. If using this probing device, it should be useful for evaluating the results in a secondary study to confirm changes.

There was one report on haptic feedbacks of soft tissues during arthroscopic probing [29]. However, the data were described in terms of numerical values that multiplied the force and moment. Additionally, the authors tried to provide feedback of ACL during pull probing, yet the results of the ACL were not included. On the other hand, since the measurement values of the probing force in the current study consisted of only the force, our method is more precise than the previous method.

There are several limitations of this study. First, the number of cadavers we enrolled was small; however, the number was not too small compared with previous biomechanical studies [11,21]. Second, this study was performed ex vivo (at room temperature and extracellular pH), and the measurement conditions differed from the in vivo environment. This is a limitation associated with most ex vivo studies before they are extrapolated to clinical use. Further studies to investigate the tension of original and/or reconstructed ACL should implement a fixation device to control the position and orientation of the probing device.

## 5. Conclusions

Using the probing device to measure the resistance force of soft tissues while probing, we measured the resistance force of the dissected ACLs and investigated its correlation with the mechanical property of the ligaments. According to the results, when a certain amount of tension (strain 16.6%) was applied, its mechanical properties were correlated with the probing force. Therefore, we believe the tension of a reconstructed ACL after fixation under real ACL reconstruction surgery can be measured by the novel probing device.

## Figures and Tables

**Table 1:** The measurements with the tensile tester.

Parameters	First Phase (Strain; 6.7%)	Second Phase (Strain, 13.3%)	Third Phase (Strain; 16.7%)
Load (N)	0.43 (SD; 0.15, Range; 0.25–0.71)	1.08 (SD; 0.26, Range; 0.79–1.64)	1.10 (SD; 0.22, Range; 1.10–1.79)
Stiffness (N/mm)	0.43 (SD; 0.15, Range; 0.25–0.71)	0.72 (SD; 0.18, Range; 0.52–1.09)	0.55 (SD; 0.09, Range; 0.44–0.71)
Young’s modulus (MPa)	0.21 (SD; 0.10, Range; 0.10–0.40)	0.26 (SD; 0.09, Range; 0.15–0.48)	0.26 (SD; 0.06, Range; 0.16–0.36)
Probing Force (Z direction (N))	0.43 (SD; 0.15, Range; 0.25–0.71)	1.08 (SD; 0.26, Range; 0.79–1.64)	1.39 (SD; 0.33, Range; 0.90–2.11)
Probing Force (Y direction (N))	0.48 (SD; 0.11, Range; 0.24–0.68)	0.57 (SD; 0.12, Range; 0.32–0.71)	0.62 (SD; 0.11, Range; 0.43–0.85)
Probing Force (resultant Z and Y (N))	0.66 (SD; 0.12, Range; 0.51–0.90)	1.23 (SD; 0.25, Range; 0.25–0.71)	1.53 (SD; 0.32, Range; 1.02–2.24)
Probing Force (X direction (N))	0.13 (SD; 0.07, Range; 0.02–0.25)	0.15 (SD; 0.10, Range; 0.03–0.35)	0.16 (SD; 0.11, Range; 0.03–0.37)
Probing Force (all resultant force) (N))	0.67 (SD; 0.13, Range; 0.53–0.93)	1.24 (SD; 0.26, Range; 0.86–1.79)	1.54 (SD; 0.33, Range; 1.03–2.26)
The amount of the increased force by the load cell while the pull probing (N)	0.48 (SD; 0.12, Range; 0.27–0.71)	0.54 (SD;0.16, Range; 0.29–0.73)	0.54 (SD; 0.14, Range; 0.37–0.79)

**Table 2:** The correlation between stiffness and Young’s modulus by the tensile tester, and the probing force by the probing device.

Parameters	First Phase (Strain; 6.7%)	Second Phase (Strain; 13.3%)	Third Phase (Strain; 16.7%)
Stiffness and probing force (only Z)	−0.10 (*p* = 0.56)	0.03 (*p* = 0.92)	0.54 (*p* = 0.11)
Stiffness and probing force (resultant Z and Y)	−0.17 (*p* = 0.56)	0.08 (*p* = 0.79)	0.53 (*p* = 0.049)
Stiffness and probing force (all resultant force)	−0.16 (*p* = 0.60)	0.14 (*p* = 0.63)	0.56 (*p* = 0.045)
Young’s modulus and probing force (only Z)	−0.16 (*p* = 0.60)	−0.12 (*p* = 0.69)	0.38 (*p* = 0.19)
Young’s modulus and probing force (resultant Z and Y)	−0.20 (*p* = 0.51)	−0.03 (*p* = 0.91)	0.40 (*p* = 0.18)
Young’s modulus and probing force (all resultant force)	−0.16 (*p* = 0.58)	−0.03 (*p* = 0.91)	0.42 (*p* = 0.14)

## Data Availability

The data presented in this study are available on request from the corresponding author.
